# Automated Detection of Normal, Atrial, and Ventricular Premature Beats from Single-Lead ECG Using Convolutional Neural Networks

**DOI:** 10.3390/s26020513

**Published:** 2026-01-12

**Authors:** Dimitri Kraft, Peter Rumm

**Affiliations:** 1 MedTec & Science GmbH, Maria-Merian-Straße 6, 85521 Ottobrunn, Germany; dimitri.kraft@ms-gmbh.de; 2 custo med, GmbH, Maria-Merian-Straße 6, 85521 Ottobrunn, Germany

**Keywords:** Premature Ventricular Contraction (PVC) detection, Premature Atrial Contraction (PAC) detection, 1D U-Net neural network, Holter monitoring

## Abstract

Accurate detection of premature atrial contractions (PACs) and premature ventricular contractions (PVCs) in single-lead electrocardiograms (ECGs) is crucial for early identification of patients at risk for atrial fibrillation, cardiomyopathy, and other adverse outcomes. In this work, we present a fully convolutional one-dimensional U-Net that reframes beat classification as a segmentation task and directly detects normal beats, PACs, and PVCs from raw ECG signals. The architecture employs a ConvNeXt V2 encoder with simple decoder blocks and does not rely on explicit R-peak detection, handcrafted features, or fixed-length input windows. The model is trained on the Icentia11k database and an in-house single-lead ECG dataset that emphasizes challenging, noisy recordings, and is validated on the CPSC2020 database. Generalization is assessed across several benchmark and clinical datasets, including MIT-BIH Arrhythmia (ADB), MIT 11, AHA, NST, SVDB, CST STRIPS, and CPSC2020. The proposed method achieves near-perfect QRS detection (sensitivity and precision up to 0.999) and competitive PVC performance, with sensitivity ranging from 0.820 (AHA) to 0.986 (MIT 11) and precision up to 0.993 (MIT 11). PAC detection is more variable, with sensitivities between 0.539 and 0.797 and precisions between 0.751 and 0.910, yet the resulting F1-score of 0.72 on SVDB exceeds that of previously published approaches. Model interpretability is addressed using Layer-wise Gradient-weighted Class Activation Mapping (LayerGradCAM), which confirms physiologically plausible attention to QRS complexes for PVCs and to P-waves for PACs. Overall, the proposed framework provides a robust, interpretable, and hardware-efficient solution for joint PAC and PVC detection in noisy, single-lead ECG recordings, suitable for integration into Holter and wearable monitoring systems.

## 1. Introduction

Premature Ventricular Contractions (PVCs) and Premature Atrial Contractions (PACs) are common types of irregular heartbeats that can signal significant cardiovascular health issues. Rapid and accurate identification of these events is critical for timely clinical intervention. Frequent PACs are notably associated with an increased risk of incident atrial fibrillation (AFib) and adverse outcomes, such as stroke [1]. Conversely, a higher PVC burden is linked to the development of cardiomyopathy [2] and structural heart disease [3].

In this paper, we propose an innovative deep learning-based approach for the detection of PVCs and PACs by reframing the task as a one-dimensional (1D) segmentation problem. We leverage the U-Net architecture [4], originally designed for image segmentation, and adapt it specifically for processing 1D electrocardiogram (ECG) signals. Our adaptation employs modern convolutional networks as encoders, enabling the model to effectively capture the inherent variability and dynamics of PVC and PAC occurrences, both within and across different individuals.

Our model reliably distinguishes between normal beats, PVCs, and PACs, even in noisy single-lead ECG recordings. Crucially, our solution is optimized to run efficiently on existing medical hardware, eliminating the need for cloud-based support, which reduces latency and enhances data privacy.

The core contributions of this work are summarized as follows:

1. We introduce a streamlined, end-to-end deep learning framework for ECG beat classification that directly outputs segmentation masks, removing the necessity for manual beat annotation.
2. Our approach eliminates the requirement for hand-crafted feature extraction by automatically learning relevant representations directly from raw ECG signals.
3. The fully convolutional nature of our architecture enables it to handle varying input signal lengths, providing enhanced flexibility in real-world scenarios.
4. Our method demonstrates robust performance under noisy conditions, effectively addressing one of the most significant challenges in practical Holter ECG monitoring.
5. We extensively evaluate our approach across multiple datasets with diverse characteristics and challenges, achieving superior performance compared to existing methodologies.

## 2. Related Work

### 2.1. PVC Detection

The use of neural networks for automated detection of cardiac arrhythmias, including PVCs and PACs, has been explored for several years. Early work by Kiranyaz et al. [5] introduced a 1D convolutional neural network (CNN) tailored for patient-specific ECG classification, notably for the detection of PVCs. Although their model demonstrated high adaptability to individual ECG patterns, it required patient-specific training, significantly restricting scalability, particularly for real-time deployment on consumer-grade devices.

Previous methods for PVC and PAC detection in Holter ECG recordings predominantly rely on precise beat segmentation followed by classification using either handcrafted features or deep learning methods. Traditional machine learning techniques often utilize annotated R-peak locations as reference points, making them vulnerable to inaccuracies from noise or incorrect beat segmentation. For instance, Mazidi et al. employed a Support Vector Machine (SVM) classifier utilizing optimized morphological and wavelet-based features, achieving an accuracy of 99.78%, sensitivity of 99.91%, and specificity of 99.37% on the MIT-BIH Arrhythmia Database [6]. Note, that they used 10% of the MIT-BIH DB as training data and a bias cannot be ruled out, even if patient-independent separation is applied. Further, only 27 records are used.

Hybrid methods, outlined by Sarshar and Mirzaei’s work [7], combine handcrafted morphological and statistical features with CNNs. On a held-out subset of 22 records from the MIT-BIH Arrhythmia Database, they report a precision of 98.6%, a sensitivity of 99.2%, and an F1 score of 98.9%. Their pipeline depends on presegmented QRS complexes (i.e., individual beats must be extracted in advance), and the degree of separation between training and test samples is not fully documented. This raises the possibility of information leakage given the heterogeneous morphologies of PVCs. Furthermore, because all features are engineered specifically for MIT-BIH data, the method’s generalizability to other ECG cohorts remains untested.

Contrasting these traditional and hybrid approaches, Kraft et al. [8] introduced a novel, fully convolutional 1D U-Net architecture designed to directly locate and classify PVCs without preliminary segmentation or manual annotation. Their method inherently captures both morphological and temporal ECG features, significantly simplifying the pipeline and reducing susceptibility to noise. Evaluated on the MIT-BIH 11 database, their method achieved a balanced-accuracy score of 98.6%, demonstrating competitive or superior results compared to previous approaches.

### 2.2. PAC Detection

Despite advancements in PVC detection, PAC detection has been comparatively underexplored, with few robust algorithms capable of concurrently addressing both arrhythmias. Early approaches relied on handcrafted features (wavelet coefficients, spectral entropy, morphological characteristics) with traditional classifiers. Recent deep learning methods show promise but face significant limitations. Wang et al. proposed [9] a 33-layer CNN enhanced with a non-local convolutional block attention module (NCBAM) achieving F1-scores above 96%. However, their evaluation approach using the first 100 beats of each record may not fully capture the variability present in longer ECG recordings. Meng et al. [10] reported 88% F1-score using lightweight transformers with SMOTE augmentation, while Yang et al. [11] achieved 72% F1-score with 1D CNNs and custom loss functions. García-Isla et al. [12] proposed beat-by-beat classification for both PAC and PVC detection. However, their method’s performance depends heavily on accurate R-peak detection, which often fails in noisy Holter ECG conditions, compromising beat segmentation and classification accuracy.

Despite these advances, numerous pitfalls remain in existing algorithms for PVC and PAC detection:

1. Dependence on Accurate R-Peak Detection: Many models require the first R-peak to be correctly detected, making them vulnerable to errors from peak detection algorithms, especially in noisy segments.
2. Assumption of a Single Arrhythmia per Segment: Some methods assume that each ECG segment contains only one type of arrhythmia, failing to account for real-world cases where multiple arrhythmias coexist. PAC and PVC can appear in the same ECG segment.
3. High Computational Cost and Slow Learning: Several deep learning models are computationally intensive, requiring significant processing power and long training times, which limits their suitability for real-time monitoring on wearable devices.
4. Limited Handling of Variable-Length ECG Data: Many models are trained on fixed-length input windows, making them inflexible when dealing with ECG recordings of different durations.
5. Real-World Noise Conditions: ECG recordings are often contaminated by baseline wander, power line interference, muscle noise, and electrode contact artifacts. Such noise can significantly affect feature extraction, causing algorithms that perform well on clean datasets to struggle in practical applications.
6. Dataset Representativeness: Demographic factors such as gender differences influence ECG characteristics including heart rate, QRS duration, and QT intervals [13,14]. Models that ignore these variations may fail to generalize across diverse populations.
7. Training and Testing Practices: Many studies use overlapping or limited datasets for both training and testing, raising concerns about the generalizability of their results. A strict separation between training and testing data is essential to properly evaluate performance on unseen and diverse ECG recordings [15].

Given these limitations, our study introduces a robust, end-to-end solution that overcomes these challenges. Our approach does not rely on separate R-peak detection, can handle mixed arrhythmias (PVC and PAC) in a single segment (varying length), and effectively processes noisy single-lead ECG signals from consumer-grade devices.

### 2.3. Beat Detection Performances

The accurate detection of normal cardiac beats is essential for the diagnosis and monitoring of cardiovascular diseases. Various algorithms have been proposed over the years, with each aiming to optimize the detection accuracy under different conditions. The Table 1 below presents a comparative assessment of several popular normal beat detection algorithms applied to the MIT-BIH dataset, a well-regarded benchmark for cardiac signal analysis. Both the complete MIT-BIH dataset and a version with VFib beats excluded are considered. Performance metrics, including Sensitivity (Se), Precision (PPV), and the F1 score, are used to gauge each algorithm’s efficacy. This table has been adapted from [16], providing a consolidated view of the advancements in this domain.

### 2.4. PVC Detection Performances

PVCs are early heartbeats originating in the ventricles of the heart. Their accurate detection is critical given their potential association with various cardiac disorders. Table 2 offers a comparative study of several prominent PVC beat detection algorithms when applied to a subset of 11 records from the MIT-BIH dataset, a renowned benchmark in cardiac signal processing. This subset provides a specific environment to evaluate the algorithms’ performance due to the unique characteristics and challenges posed by PVC beats. The performance metrics included are Sensitivity (Se), Precision (PPV), and the F1 score. This table, adapted from [16], enables readers to comprehend the current state of the art in PVC beat detection and the relative efficacy of different methods.

### 2.5. PAC Detection Performances

PACs are early heartbeats that originate from the atria. Although often benign, their occurrence can indicate underlying atrial abnormalities and may precede more serious arrhythmias. Therefore, accurate PAC detection is essential for early diagnosis and risk assessment in clinical practice.

Recent studies have investigated a variety of automated techniques, ranging from linear discriminant classifiers that use RR interval and morphological features to advanced methods incorporating wavelet-based analyses. Table 3 presents a concise overview of these approaches, including performance metrics such as sensitivity, positive predictive value (PPV), and false positive rate (FPR) across different databases. This comparison underscores both the progress achieved and the remaining challenges in developing reliable PAC detection algorithms.

## 3. Methods

In the methodology section of this paper, we delve into the specific techniques utilized for the detection and differentiation of normal, PVC and PAC beats. We are building upon our previous work [8]. Given the critical implications these heart rhythms have in clinical practice and patient care, the accuracy and efficiency of these detection algorithms are paramount. Our methodological approach incorporates a broad range of computational tools and techniques, each tailored to handle the specific characteristics of normal heartbeats, PVCs and PACs. These include, but are not limited to data preparation, signal preprocessing, machine learning algorithms, and evaluation metrics.

### 3.1. Overview

In this study, we employ a 1D U-Net architecture [4] for end-to-end detection of PVCs and PACs. The model incorporates a ConvNeXt V2 encoder [34] and four lightweight decoder blocks, each consisting of an upsampling layer, a 1D convolutional layer, Layer Normalization [35], and a LeakyReLU activation [36]. The network starts with 16 filters in the first layer, with the number of filters doubling after each encoder stage. All ConvNeXt V2 blocks use a kernel size of 7 with a dilation rate of 1.

The encoder begins with a stem layer composed of a 1D convolution (kernel size = 4, stride = 4) followed by Layer Normalization. This is followed by three downsampling stages, each consisting of Layer Normalization and a 1D convolution (kernel size = 2, stride = 2), which progressively reduce the temporal resolution while increasing the feature dimensionality.

Our training data came from the Icentia 11k dataset (https://physionet.org/content/icentia11k-continuous-ecg/ (accessed on 7 January 2026)) and a custom dataset we curated, which includes roughly 7500 single-lead ECG windows lasting between 10 and 30 s. This custom dataset, designed to capture challenging real-world scenarios, is not publicly available. We targeted the detection of normal beats, PVCs, and PACs. For this purpose, we created a segmentation mask where:

- Class 1 marks normal beats (a 200 ms window around the R peak);
- Class 2 marks PVCs (from 100 ms before to 150 ms after the R peak);
- Class 3 marks PACs (a 200 ms window around the R peak).

**Figure 1:**
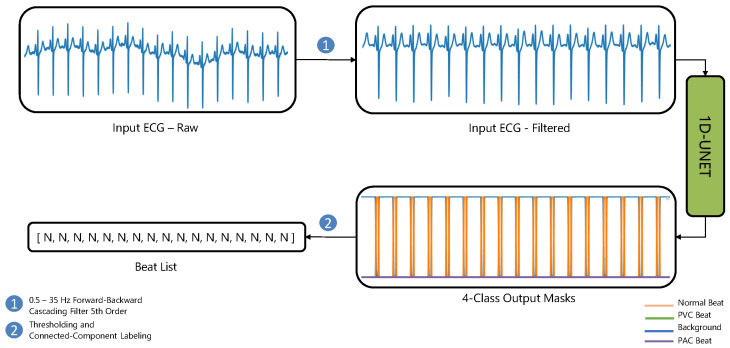
Diagram illustrating our method for Normal, PVC and PAC detection with single-lead ECGs. For multi-lead ECG analyses, each lead is processed independently, with beat lists from all leads subsequently integrated via a majority and priority voting mechanism.

The background is labeled as class 0. Figure 1 illustrates our overall approach using a 1D U-Net.

### 3.2. Data Preparation

Accurate and effective arrhythmia classification relies heavily on high-quality ECG data and appropriate preprocessing techniques [37]. In this study, we utilized two datasets for training: (1) the Icentia11k dataset (https://physionet.org/content/icentia11k-continuous-ecg/ (accessed 7 January 2026)) and (2) a custom dataset (not publicly available). Each dataset was subjected to specific preprocessing steps to ensure consistency and enhance model performance. The primary objective of the data preparation process was to isolate clinically relevant arrhythmic events while maintaining a balanced representation of normal and abnormal beats.

For both datasets, 20-s ECG segments were extracted to capture essential temporal patterns associated with PACs and PVCs. Targeted filtering techniques, including Butterworth filtering and power line noise removal, were applied to suppress noise and baseline wander. To address inherent class imbalance, particularly for arrhythmic events, an oversampling strategy was employed for minority classes to create a more balanced and representative training dataset.

The following subsections provide a detailed overview of the data selection, preprocessing, and segmentation strategies applied to each dataset.

#### 3.2.1. Preprocessing the Icentia11k Dataset

The Icentia11k dataset contains millions of annotated PAC and PVC beats. To better capture complex arrhythmic patterns, including couplets, runs, supraventricular tachycardia, and ventricular tachycardia, we selected ECG segments containing at least three consecutive PAC or PVC beats. From these runs, 20-s windows were extracted to ensure the inclusion of diverse PAC and PVC combinations during model training. These segments were further oversampled during training to mitigate class imbalance.

#### 3.2.2. Custom Dataset Preparation

ECG recordings in the custom dataset were divided into non-overlapping 20-s segments, with a corresponding segmentation mask generated for each segment as previously described. Signals shorter than 20 s were zero-padded to achieve the required duration. Amplitude normalization was intentionally omitted; instead, a series of preprocessing steps was applied. These included a forward–backward high-pass Butterworth filter with a cutoff frequency of 0.5 Hz and a power line noise filter. All signals were subsequently downsampled to 125 Hz using linear interpolation. These preprocessing steps were applied to the entire signal prior to segmentation and were consistently used across all datasets.

**Figure 2:**
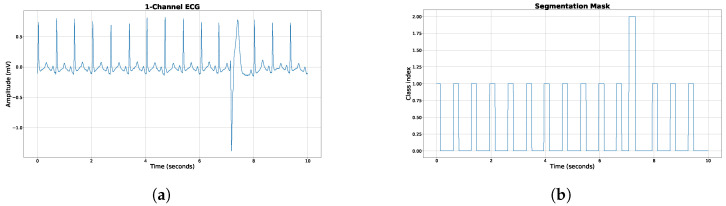
Example ECG from the MIT DB and the corresponding label mask. (**a**) Example ECG from the MIT Arrhythmia dataset. A single PVC is located at around second 7. (**b**) The corresponding mask containing background, normal beats and PVC encoded with 0, 1 and 2, respectively.

Only samples containing normal, PVC, or PAC beats were included in the dataset. Windows containing beats that could not be reliably classified during labeling were excluded. Figure 2 illustrates a representative 10-s ECG segment along with its corresponding segmentation mask.

The segmentation mask was constructed as follows. For normal beats, a label of class index 1 was assigned from 100 ms before to 100 ms after the R-peak. For PVC beats, a label of class index 2 was assigned from 100 ms before to 150 ms after the R-peak to capture the extended morphology of PVCs. All remaining regions were labeled as background (class index 0), as shown in Figure 2b. The resulting training dataset comprised approximately four million 20-s segments. To further address class imbalance, oversampling of PVC and PAC segments was applied during training.

#### 3.2.3. Model Architecture

**Figure 3:**
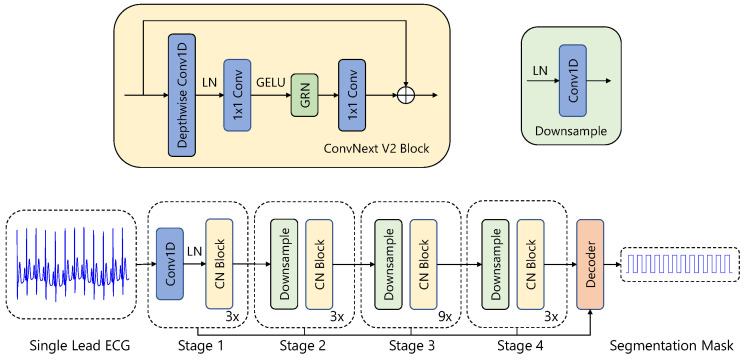
Architecture of the proposed neural network for PAC and PVC detection from single-lead ECG signals. The encoder-decoder structure is based on ConvNextv2 [34] adapted for 1D temporal data. The encoder consists of four stages with progressively increasing receptive fields. Each stage contains ConvNext V2 blocks (beige boxes) and downsampling operations (light green boxes). The ConvNext V2 block architecture (detailed in upper left) comprises depthwise convolution, layer normalization (LN), pointwise convolutions (1 × 1 Conv), GELU activation, and Global Response Normalization (GRN) with residual connection (indicated by ⊕). Blue boxes represent convolutional layers. The decoder (coral-colored box) processes the encoded representations to generate the final segmentation mask identifying arrhythmic events. Black arrows indicate forward propagation through the network.

Our 1D U-Net (see Figure 3) follows the standard U-Net design, comprising an encoder (contracting path), a bottleneck, and a decoder (expanding path):

- Encoder: The encoder extracts deep hierarchical features from the ECG signal. We use a ConvNext V2 encoder [34], which starts with a stem layer consisting of a 1D convolution (kernel size = 4, stride = 4) followed by LayerNorm [35]. Three additional downsampling layers, each combining LayerNorm and a 1D convolution (kernel size = 2, stride = 2), progressively reduce the temporal resolution while increasing feature dimensionality.
- Feature Extraction Blocks: At each level of the encoder, feature extraction is performed using ConvNext V2 blocks, which include:
  – Depthwise Convolutions (kernel size = 7) to capture temporal dependencies in ECG signals.
  – Layer Normalization and GELU Activation for stable training and improved non-linearity.
  – Pointwise Convolutions, expanding and reducing feature dimensions to enrich feature representations.
  – Global Response Normalization (GRN) to encourage feature diversity.
  – Residual Connections to retain critical information and stabilize gradient flow.
- Bottleneck Layer: The bottleneck serves as the transition between the encoder and decoder, maintaining high-level feature abstraction. Unlike conventional U-Nets that rely on two standard convolutional layers, we employ depthwise-separable convolutions with a kernel size of 7 to balance efficiency and performance.
- Decoder: The decoder reconstructs the segmentation mask by progressively upsampling the feature maps. Each decoder block consists of:
  – An upsampling layer to restore the temporal resolution.
  – A convolutional layer to refine features.
  – Layer Normalization and LeakyReLU activation to stabilize training and prevent saturation effects.
  – Skip connections that fuse high-resolution encoder features with decoder outputs, aiding in precise beat localization.

#### 3.2.4. Final Output

The final layer applies a 1D convolution (kernel size = 1) to generate a segmentation mask. The output shape is L×C, where *L* is the input ECG length and *C* is the number of classes (normal, PVC, PAC, and background).

Our approach is fully convolutional, meaning it can process input ECGs of varying lengths without requiring fixed-sized segments. Additionally, our model directly outputs a segmentation mask, eliminating the need for preliminary beat detection or feature engineering.

#### 3.2.5. Hyperparameter Selection

The model was optimized using the Dice loss function adapted for 1D segmentation tasks [38]. Training employed the AdamW optimizer ($\beta_{1}=0.9$, $\beta_{2}=0.999$) without weight decay [39], with an initial learning rate of 0.001. A one-cycle learning rate scheduler was used, with the maximum learning rate set to 0.001. All hyperparameters were selected through an extensive grid search.

### 3.3. Data Augmentation

During the training phase, a strategic dynamic augmentation of data was undertaken to promote a higher degree of model generalizability and resilience against variations in real-world scenarios. This augmentation process incorporated the random scaling of signal amplitudes, the infusion of random Gaussian, pink and brown noise, and the induction of minor baseline shifts. Notably, we refrained from employing other prevalent augmentation procedures, such as signal masking, temporal shifting, mixup [40], and cutmix [41].

**Figure 4:**
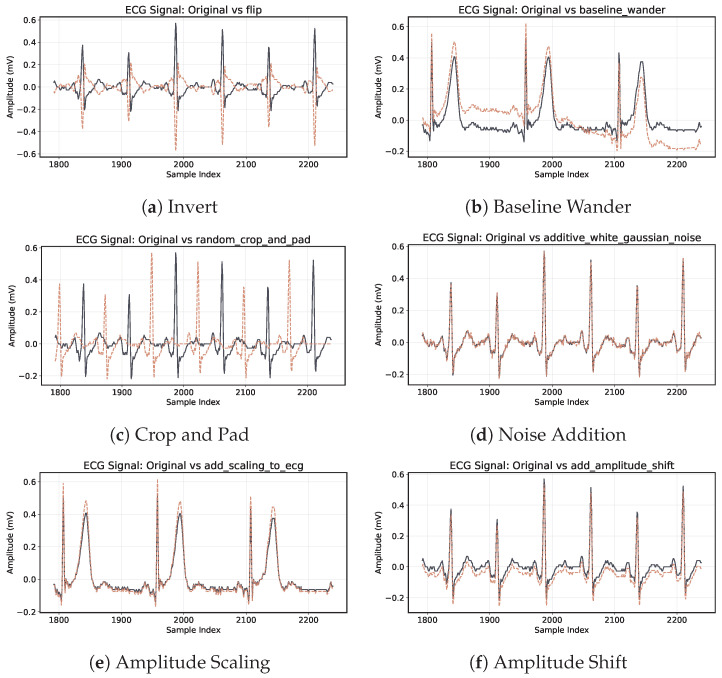
Visualization of ECG augmentation techniques. The black signal represents the ECG before augmentation. The dashed signal represents the ECG after augmentation. Note that for crop and pad, the segmentation mask is modified to adjust for stretching and compression in time.

To evaluate the effect of data augmentation, we apply a combination of the following transformations dynamically during training:

- Scaling the amplitude with a probability of p=0.75 and scaling factor of [0.6…1.4] (Figure 4e).
- Offset the amplitude with a probability of p=0.75 and a offset value of [−0.1…0.1] (Figure 4f).
- Adding gaussian, brown or pink noise with a probability of p=0.75 (Figure 4d).
- Adding baseline wander with a probability of p=0.75 (Figure 4b).
- Adding random spikes with a probability of p=0.25.
- Crop and Pad with a probability of p=0.25 (Figure 4c).
- Invert the ECG by multiplying by −1 with a probability of p=0.35 (Figure 4a).

Although data augmentation doesn’t invariably lead to enhanced performance in practical scenarios, certain methods can adversely affect outcomes, as highlighted by Raghu et al. [42] in the context of AFib detection. However, based on our trials, the signal transformations discussed previously proved optimal for our distinct task and dataset. This aligns with Rahman et al.’s systematic review [43] on ECG signal data augmentation.

### 3.4. Post-Processing

The post-processing is carried out as follows: First, a QRS Mask is computed by subtracting the background mask from 1 since we use softmaxed values:

QRSMask=1−BackgroundMask.

Using this QRS mask, contiguous regions (objects) where the mask exceeds a predefined threshold of 0.9 for a minimum duration are identified. Each detected object is then classified by computing the mean values of the Normal, PVC, and PAC masks within that region. The object is assigned a beat label based on these mean values.

When merging detections across multiple channels, a majority voting system is applied along with a hierarchical rule: PVC beat detections are given the highest priority, followed by PAC and then Normal beats.

### 3.5. Training Data

We utilize two different datasets as training data. The first one is a custom 3-lead ECG dataset collected from a wide variety of subjects. The second dataset is the Icentia11k dataset, consisting of single channel ECG data from 11,000 subjects, a large dataset for unsupervised training.

#### 3.5.1. Pretraining on Icentia11k

We first pretrained the model on Icentia11k for PAC and PVC detection. We then applied the model to generate new training data sampled from our custom dataset. We corrected wrong annotations and use this new data in our custom training dataset.

#### 3.5.2. Custo Med Training Dataset

**Figure 5:**
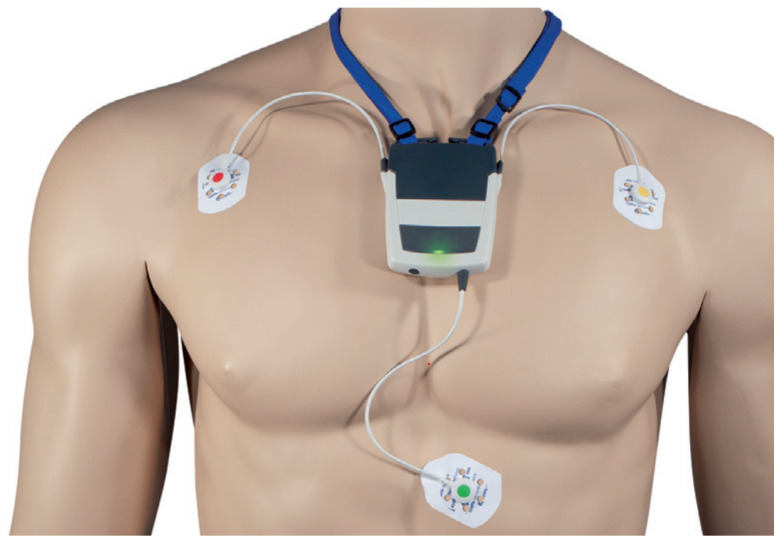
Custo med flash 500/510 3-Channel Holter used for data acquisition of our custom dataset.

We utilized a custo med flash 500/510 3-Channel Holter (see Figure 5), with a sampling frequency of 125 Hz and 5.6 microvolt/Bit resolution with 10 bit resolution.

This data were obtained in an anonymized form from one of our clients. As such, we do not possess information regarding the age and sex of the individuals associated with the electrocardiograms. For the training data, approximately 1000 ECGs were employed, albeit not all from unique patients. From these ECGs, we generated between 3 to approximately 30 snippets of varying lengths (ranging between 10 s to around 120 s) which resulted in 7500 ECG samples. These ECG snippets underwent careful evaluation, with corrections made to annotations as necessary to ensure accuracy. In addition, we utilized 36 different 24-h ECG recordings taken from 36 unique patients for an extended, long-term monitoring evaluation. The design of this dataset was aimed at focusing on QRS and PVC classes specifically, thus only these were annotated (see Table 4).

#### 3.5.3. Icentia11k Dataset Overview

The Icentia11k dataset comprises 11,000 single-lead (modified lead I) ECG recordings (250 Hz, 16-bit) from CardioSTAT monitors in Ontario, Canada [44]. Beats were auto-detected and manually labeled (beat type and rhythm) via full-disclosure review by Icentia technologists, with senior approval (see Table 5). Mean patient age was 62.2 ± 17.4 years. From 2 billion normal and 17 million PVC beats, we (1) subsampled 1% of normal segments (no PAC or PVC), (2) retained all segments with ≥1 PVC/PAC, (3) enriched runs of ≥3 PVC/PAC, and (4) created alternating PAC–PVC sequences, then oversampled both sets to balance pathologies.

### 3.6. Validation Data

We use the CPSC2020 dataset [45] as our validation set during training. We track the F1 scores for PAC and PVC and employ early stopping based on these metrics to prevent overfitting.

#### CPSC2020 Dataset Overview

The CPSC2020 dataset (3rd China Physiological Signal Challenge 2020) (https://opensz.oss-cn-beijing.aliyuncs.com/ICBEB2020/file/TrainingSet.zip (accessed on 7 January 2026)) comprises ten continuous 24 h single-lead ECG recordings from arrhythmia patients (including some with atrial fibrillation) for training, plus a similarly structured test set withheld for scoring. Signals are sampled at 400 Hz and stored as MATLAB v4 “.mat” files containing the raw ECG alongside annotation files for PVCs and PACs (see Table 6). Each 23–26 h recording contains approximately 70,000 to 140,000 heartbeats (PVCs: 0–19,000 per recording; PACs: up to 9000). Annotations for normal beats are not provided. This dataset is intended to serve as a more challenging benchmark compared to standard MIT datasets such as the MIT–BIH Arrhythmia Database. We used a SOTA QRS Detector [8] to create normal beat annotations.

### 3.7. Test Data

The test data consists of publicly available and private datasets. No testing data is included in the training data, ensuring good generalizability of our model. We utilize several standard datasets including MIT, MIT-11 subset, AHA, NST (for noisy conditions evaluation), and our own collected datasets (CST and CST Strips).

#### 3.7.1. Test Dataset Descriptions

MIT-BIH Supraventricular Arrhythmia Database (SVDB)—Open-source database available on PhysioNet (https://physionet.org/content/svdb/1.0.0/ (accessed on 7 January 2026)) with 2-lead ECG recordings at 128 Hz, approximately 30 min duration each. Rich in supraventricular events with high variety of PAC occurrences including bigeminy, trigeminy, and atrial runs. Records 841, 857, 822, 867, 866 excluded due to wrong annotations or containing ECG with atrial flutter.

American Heart Association Database (AHA)—80 two-channel ambulatory ECG recordings from late 1970s-early 1980s (https://www.ecri.org/(accessed on 7 January 2026)), digitized at 250 Hz with 12-bit resolution over 10 mV range. Contains 30-min annotated segments classified by ventricular ectopy severity ranging from ’no ventricular ectopy’ to ’ventricular flutter/fibrillation’.

MIT-BIH Arrhythmia Database (ADB) [46]—48 two-channel ambulatory ECG recordings (half-hour each) from 47 subjects, digitized at 360 samples/second with 11-bit resolution over 10 mV range. Each record annotated independently by two or more cardiologists. We utilize 44 samples for evaluation, totaling 24.07 h of data.

Noise Stress Test Database (NST) (https://physionet.org/content/nstdb/ (accessed on 7 January 2026))—15 half-hour recordings with varied Signal-to-Noise ratios (24 dB to −6 dB) generated from clean MIT-BIH records with artificially added noise to test algorithm robustness under different noise conditions [47].

Custo Med Test Dataset (CST Strips)—Our collected dataset (not publicaly available) consisting of 627 records of 10–30 s ECG segments with focus on noisy, hard-to-classify PVC beats, couplets, triplets, and salves from different subjects.

#### 3.7.2. Test Dataset Summary

Table 7 presents a overview of beat counts across all utilized datasets.

The combined dataset provides a comprehensive evaluation framework with over 529,000 annotated beats spanning various recording conditions, noise levels, and arrhythmia complexities. All datasets focus primarily on Normal and PVC beat detection, with selected datasets including PAC annotations. The *NST* dataset, while not included in beat counts as it derives from MIT records, serves specifically for noise robustness validation across different SNR conditions.

## 4. Results

The quantitative results on our test data are outlined in detail in Table 8. We utilized Sensitivity (Se) and Precision (Pr) to assess the performance of our model. We surpass the state of the art on the MIT 11 dataset in PVC detection with a additional percentage point in sensitivity compared to our prior work [8] and show stable performance in QRS and PVC detection across all data sets.

The metrics are calculated as follows:

(1) $$Pr=\frac{TP}{TP+FP};\;Se=\frac{TP}{TP+FN};$$

### 4.1. Error Analysis

#### MIT ADB

In a nonparametric bootstrap of 1000 iterations (Table 9), overall QRS detection is virtually flawless (sensitivity = 0.989 [95% CI 0.978–0.999], precision = 0.999 [0.998–0.999], $F_{1}$ = 0.994 [0.988–0.999]), with CI widths ≤ 0.010 indicating extreme stability. PVC classification attains high precision (0.977 [0.960–0.990]) and good sensitivity (0.897 [0.825–0.973], CI width 0.148), yielding $F_{1}$ = 0.935 [0.893–0.974]. By contrast, PAC performance is more variable (sensitivity = 0.892 [0.726–0.973], precision = 0.714 [0.433–0.882], $F_{1}$ = 0.789 [0.557–0.917], CI width up to 0.360), reflecting class imbalance. Support-weighted averages mirror these trends (PVC ${\mathrm{precision}}_{w}$ = 0.969, ${\mathrm{sensitivity}}_{w}$ = 0.977; PAC ${\mathrm{precision}}_{w}$ = 0.928, ${\mathrm{sensitivity}}_{w}$ = 0.714). These results underscore nearly perfect beat detection overall, excellent PVC identification, and the need for additional PAC examples or imbalance-mitigation strategies to improve and stabilize PAC classification.

### 4.2. Evaluation Method

The objective of our evaluation is generate a list of Normal, PVC and PAC beat locations for each recording that align with the ground truth annotations. For every reference annotation, there should be a corresponding predicted annotation within a 150 ms interval centered around it. Note that reference annotations present in the first or last 0.2 s of the recording are disregarded. Any detected beat should fall within 150ms of its reference annotation. We mapped the MIT-BIH annotation codes to our three beat classes as follows according to [48]:

- Normal: NORMAL, LBBB, RBBB, BBB;
- PAC: NPC, APC, SVPB, ABERR, NESC, AESC, SVESC;
- PVC: RONT, PVC, VESC.

### 4.3. Model Output

**Figure 6:**
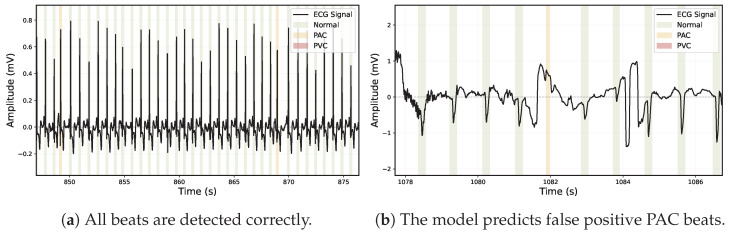
Example ECG from the custom test dataset, segmented into Normal (green) and PAC (orange) beats. No PVC beats are present in these figures.

The output of the system is a series of classifications for each timestamp in the input ECG data—as either a background, Normal, PVC or PAC beat (see Figure 6). The model outputs for a given variable-length ECG a corresponding equal length segmentation mask. The minimum length of the ECG signal length is 400 input timestamps which in our case (signal sampled at 125 Hz) equals to 400/125 = 3.2 s of input data due to the kernel size of 7, the convolutional stride of 2, same padding and a dilation of 1.

### 4.4. Learned Filters

**Figure 7:**
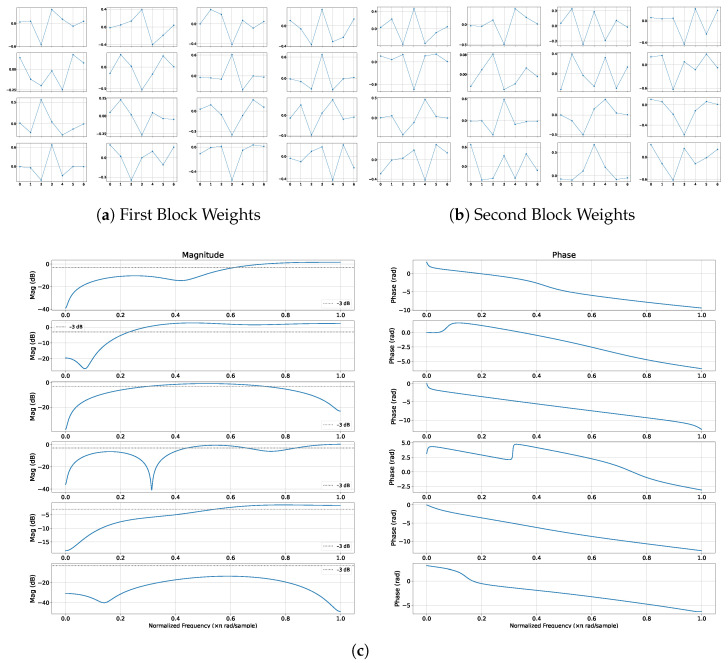
Learned 1 × 7 Kernel Weights for the Convolutional Layer from Stage 1. (**c**) Magnitude (dB) and phase (rad) responses of the first six depthwise filters from Stage 1 of a ConvNeXt-V2 block. The filters exhibit low-pass, high-pass, and band-pass characteristics; the grey dotted line marks the −3 dB cutoff.

Figure 7a shows the learned 1×7 depthwise kernels from Stage 1 in two different blocks. Neither block contains any flat (constant) kernels; instead, all filters exhibit structured, frequency-selective shapes.

#### Filter Analysis

**Figure 8:**
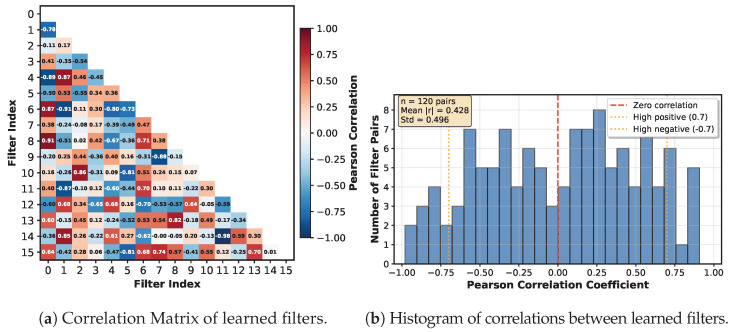
Histogram and Correlation Matrix of the learned filters of the first layer of the neural network.

This section examines the learned filters to determine whether they detect unique patterns in the signal. If filters correlate too much, they learn the same patterns. If the correlation coefficient is strongly negative, the filters learn opposite patterns. If there is no correlation at all, the filters identify unique patterns. Figure 8a show the general correlation coefficient between different filters of the first neural network layer (stem). The histogram in Figure 8b shows that only a few kernels have correlations greater than 0.7, indicating that the kernels in the first layer of the neural network learned representative and unique patterns.

## 5. Model Interpretability

**Figure 9:**
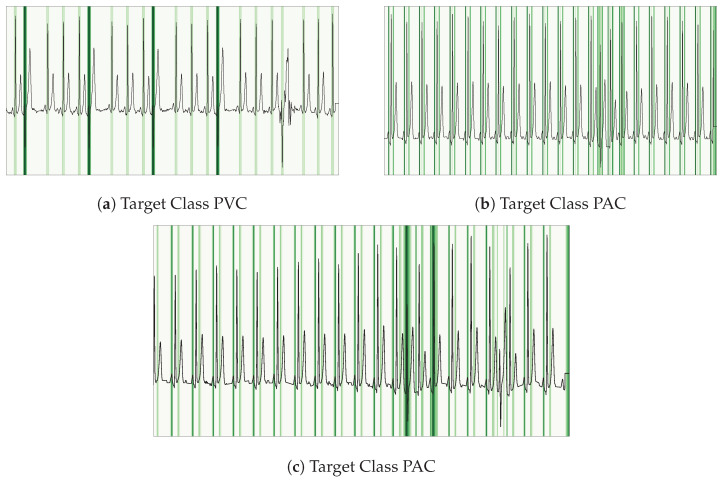
LayerGradCAM saliency maps on ECG waveforms (CPSC2020 validation), highlighting the features most influential to the network’s final decoder convolutional layer. Darker green indicates higher attribution.

To clarify our network’s decision process on ECG data, we apply Layer-wise Gradient-weighted Class Activation Mapping (LayerGradCAM) [49]. This technique produces a saliency heatmap over the input time series, where darker green regions indicate higher contributions to the model’s final segmentation output (see Figure 9).

In clinical ECG analysis, understanding why the model reaches a particular decision is as important as the decision itself. LayerGradCAM highlights the ECG segments, including specific heartbeats or waveform components, that most influence the network’s predictions. This approach improves interpretability and helps build confidence among end users.

Our key observations from the CPSC2020 validation set are:

1. QRS Complex Dominance: The model consistently assigns the highest attribution to the QRS complex across most arrhythmia classes.
2. Contextual Awareness in PVCs: For PVC beats, the network’s focus extends beyond the target beat, incorporating information from adjacent beats.
3. P-Wave Salience in PACs: In PAC classifications, the P-wave emerges as the main feature influencing the model’s decision. This characteristic may lead to false positives when the P-wave is obscured by noise or appears far from the QRS complex, as in cases of atrioventricular (AV) block, resulting in incorrect PAC detections.

Other attribution methods, including Integrated Gradients [50] and DeepLIFT [51], provide complementary insights. However, LayerGradCAM’s spatially resolved maps are especially intuitive for time-series data such as ECGs.

## 6. Discussion

The performance metrics summarized in Table 8 provide detailed insight into the strengths and limitations of our model across diverse datasets. For QRS detection, our model achieves exceptional results on most datasets, with sensitivity and precision values approaching 0.999 on the MIT DB, MIT 11, CST STRIPS, and SVDB. These results demonstrate the model’s robust capability to identify QRS complexes under varied conditions. The MIT NST dataset shows a lower sensitivity (0.870), likely reflecting the challenges posed by severe noise; however, its precision remains high (0.991). This suggests that the model refrains from identifying beats in highly noisy segments, thereby avoiding false detections.

For PVC detection, the model also exhibits strong performance. The MIT 11 dataset achieves the highest sensitivity (0.986) and precision (0.993), resulting in an F1-score of 0.989, while the MIT DB shows similarly high values (0.978 and 0.956, respectively). A slight decline is observed in the AHA dataset (sensitivity of 0.820 and precision of 0.956), which may be attributed to differences in annotation quality or to the presence of additional complexities such as pacemaker signals and overlapping arrhythmic patterns. In particular, records containing Ventricular Flutter and Fibrillation (those beginning with 82XX) present a challenge for PVC detection within this dataset. We suggest that such records be excluded to ensure a fair evaluation of PVC detection performance.

PAC detection results show greater variability and tend to be less robust overall. For example, the MIT DB and SVDB datasets yield moderate results, with sensitivities of 0.747 and 0.797 and precisions of 0.910 and 0.751, respectively. Nonetheless, our approach achieves higher precision than that reported by García et al. [12], who obtained a PPV of 0.30 and a sensitivity of 0.92, resulting in an F1-score of 0.45 compared to our value of 0.72. Furthermore, we do not employ cross-validation, as our model is not trained on the SVDB. Even when cross-validation with a patient-wise split is applied, the approach fails to fully represent general performance due to inherent biases in the database, such as identical sensors and recurring pathologies across patients.

Overall, these findings highlight the robustness of QRS detection across multiple datasets and reveal moderate variability in PVC detection. The reduced performance observed in the NST DB and AHA DB suggests that the model does not perform optimally under strong noise conditions, indicating potential directions for future work in noise resilience and algorithm optimization. We have also refined our model architecture and training pipeline compared to our previous work [8].

## 7. Limitations

While our study provides novel insights into the performance of PAC and PVC detection and adds value to the current literature, it is not without its limitations which offer avenues for future work.

1. Absence of Attention Mechanism: Our model does not include explicit attention modules. While attention mechanisms can help highlight relevant temporal regions in ECG signals, recent studies show only limited performance improvements for single-lead beat classification tasks [52]. Given our focus on real-time and hardware-efficient inference, we prioritized architectural simplicity over marginal accuracy gains. Future work could nonetheless explore lightweight attention or adaptive context modules for multi-lead or rhythm-level analyses.
2. Lack of Self-supervised Pretraining: Our study did not exploit self-supervised pretraining on multiple datasets. Such pretraining could improve robustness and generalizability by exposing the model to a wider range of ECG morphologies and noise conditions before supervised fine-tuning.
3. No Multi-task Learning: The current framework is trained solely for beat-wise classification of normal, PVC, and PAC beats. We do not jointly optimize related tasks, such as rhythm classification, noise estimation, or signal quality assessment. A multi-task learning setup could encourage the network to learn richer shared representations and might improve both detection accuracy and robustness.
4. Limited Classification: The scope of our model was confined to the detection of normal, PVC, and PAC beats. Although this focus has its merits, the model’s utility could be enhanced by expanding its classification capabilities to detect other types of cardiac events, for example fusion beats or ventricular flutter episodes.
5. Size of the Test Datasets: Our test datasets were not particularly large. Larger and more diverse test cohorts would provide a more robust estimation of the model’s performance and its ability to generalize to unseen data and rare arrhythmias.
6. Single-channel Model: Our model was designed to work with single-channel ECG signals. While this design decision simplifies the model and its input requirements, it may limit the ability to detect cardiac events that are better characterized using multichannel ECG recordings. Future research could investigate the benefits of extending the architecture to multi-lead inputs.

## 8. Conclusions

PVC detection performance was competitive with the state of the art, achieving sensitivities from 0.820 (AHA DB) to 0.986 (MIT 11 DB) and precision values up to 0.993 (MIT 11 DB). These results demonstrate that, despite the inherent variability of PVC morphology and the challenges posed by high-noise conditions in datasets such as NST and AHA, the model maintains robust and balanced detection of true PVC events.

In contrast, PAC detection performance was more variable, reflecting the difficulty of reliably identifying subtle atrial activity in noisy, single-lead recordings. The literature consistently notes that PAC annotation itself is challenging and prone to inter-observer disagreement. Llamedo and Martinez [33], for instance, reported substantial variability among expert annotators when labeling PACs. Furthermore, widely used databases such as the MIT-BIH Arrhythmia Database contain inconsistencies in less common arrhythmia labels, contributing to potential mislabeling and uncertainty in performance assessment.

Overall, these findings confirm the reliability of our QRS and PVC detection while highlighting remaining challenges in PAC identification under noisy conditions. Future work will focus on improving PAC sensitivity through enhanced data curation, noise-robust training strategies, and model optimization for single-lead wearable applications.

## Figures and Tables

**Table 1:** Comparison for QRS detection algorithms on MIT (VFib excluded)—adapted from [16].

Algorithm	Dataset	Se (%)	PPV (%)	F1
Pan and Tompkins [17]	MIT-BIH	99.76	99.56	99.66
Christov [18]	MIT-BIH	99.74	99.65	99.69
Chiarugi et al. [19]	MIT-BIH	99.76	99.81	99.78
Chouakri et al. [20]	MIT-BIH	98.68	97.24	97.95
Elgendi [21]	MIT-BIH	99.78	99.87	99.82
BeatLogic [16]	MIT-BIH	99.60	99.78	99.69
Liu et al. [22]	MIT-BIH	99.00	99.20	99.10
He et al. [23]	MIT-BIH	99.56	99.72	99.64
Kraft et al. [8]	MIT-BIH	**99.94**	**99.74**	**99.84**
Martinez et al. [24]	MIT-BIH VFib excluded	**99.80**	**99.86**	**99.83**
Arzeno et al. [25]	MIT-BIH VFib excluded	99.68	99.63	99.65
Zidelmal et al. [26]	MIT-BIH VFib excluded	99.64	99.82	99.73
BeatLogic [16]	MIT-BIH VFib excluded	99.60	99.90	99.75

**Table 2:** Comparison for PVC beat detection algorithms on MIT 11 records subset—adapted from [16].

Algorithm	Dataset	Se (%)	PPV (%)	F1
de Chazal et al. [27]	MIT-BIH 11	77.5	90.6	83.5
Jiang and Kong [28]	MIT-BIH 11	94.3	95.8	95.0
Ince et al. [29]	MIT-BIH 11	90.3	92.2	91.2
Kiranyaz et al. [5]	MIT-BIH 11	95.9	96.2	96.0
Zhang et al. [30]	MIT-BIH 11	97.6	97.6	97.6
BeatLogic [16]	MIT-BIH 11	**97.9**	98.9	98.4
Kraft et al. [8]	MIT-BIH 11	97.6	**99.4**	**98.6**
Liu et al. [22]	MIT-BIH (22 Records)	**91.6**	**95.6**	**93.6**
Cai et al. [31]	MIT-BIH (44 Records)	87.5	92.5	90.1
Kraft et al. [8]	MIT-BIH (44 Records)	**96.5**	**92.7**	**94.5**

**Table 3:** Summary of PAC Detection Performance.

Study	Method, Database & Performance Summary
De Chazal et al. [27]	**Method:** RR intervals and morphological features with a linear discriminant classifier. **Database:** MITBIH Arrhythmia Database (DB1/DB2). **Performance:** Sensitivity = 75.9%, PPV = 38.5%, FPR = 4.7%.
Llamedo and Martinez [32]	**Method:** Linear discriminant classifier with RR interval and wavelet-based morphological features. **Database:** DS2-Test set & full MITBIH. **Performance:** DS2-Test: Sensitivity (SVEB) = 77%, PPV = 88%; Full MITBIH: Sensitivity = 61%, PPV = 73%.
Llamedo and Martinez [33]	**Method:** Extended classifier combining RR and DWT features, operable in automatic or assisted mode. **Database:** Multiple public databases (including SVDB and LTSTDB). **Performance: Automatic Mode:** MITBIH: Sensitivity = 76%, PPV = 43%; SVDB: Sensitivity = 47%, PPV = 50%; LTSTDB: Sensitivity = 50%, PPV = 8%. **Assisted Mode:** MITBIH: Sensitivity = 89%, PPV = 88%; SVDB: Sensitivity = 74%, PPV = 79%; LTSTDB: Sensitivity = 51%, PPV = 58%.

**Table 4:** Count and description of ECG beat types—Custo med training dataset.

Symbol	Beat Description	Count
N	Normal	3,361,174
V	Premature ventricular contraction	163,592
S	PAC	not labeled

**Table 5:** Count and description of ECG beat types— Icentia11k.

Symbol	Beat Description	Count
N	Normal	2,061,141,216
S	Premature or ectopic supraventricular beat	19,346,728
V	Premature ventricular contraction	17,203,041
Q	Undefined: Unclassifiable beat	676,364,002

**Table 6:** Count and description of ECG beat types—CPSC 2020 DB.

Symbol	Beat Description	Count
N	Normal	945,187
V	Premature ventricular contraction	42,075
S	Premature atrial contraction	17,535

**Table 7:** Combined ECG beat counts across all test datasets.

Dataset	Normal	PVC	PAC	Total	Duration
SVDB	162,100	9930	12,090	184,120	∼30 min each
AHA	174,260	16,296	0 *	190,556	30 min segments
ADB	100,718	7009	3026	110,753	24.07 h total
CST Strips	39,133	4576	0 *	43,709	10–30 s segments
Total	476,211	37,811	15,116	529,138	–

* Note: AHA and CST Strips databases are not differentiate supraventricular ectopic beats from normal sinus beats.

**Table 8:** Mean performance metrics for arrhythmia detection. PAC metrics for AHA and CST Strips databases: not determinable (n.d.) owing to dataset limitations.

Dataset	QRS_*Se*_	QRS_*Pr*_	PVC_*Se*_	PVC_*Pr*_	PAC_*Se*_	PAC_*Pr*_
ADB	0.999	0.997	0.978	0.956	0.747	0.910
ADB 11	0.999	0.999	0.986	0.993	0.355	0.713
AHA	0.994	0.996	0.820	0.956	n.d.	n.d.
NST	0.870	0.991	0.868	0.986	0.539	0.915
CST STRIPS	0.996	0.997	0.925	0.974	n.d.	n.d.
SVDB	0.999	0.999	0.897	0.946	0.797	0.751
CPSC2020	0.989	0.999	0.882	0.903	0.734	0.855

**Table 9:** Bootstrap results (1000 iterations) for MIT ADB performance metrics.

Metric	Mean	95 % CI	Std. Dev.
Premature Ventricular Contractions (PVC)			
Sensitivity	0.897	(0.825–0.973)	0.039
Precision	0.977	(0.960–0.990)	0.008
F1-Score	0.935	(0.893–0.974)	0.021
Premature Atrial Contractions (PAC)			
Sensitivity	0.892	(0.726–0.973)	0.071
Precision	0.714	(0.433–0.882)	0.122
F1-Score	0.789	(0.557–0.917)	0.100
Normal Beats (N)			
Sensitivity	0.987	(0.981–0.993)	0.003
Precision	0.997	(0.995–0.998)	0.001
F1-Score	0.992	(0.988–0.995)	0.002
QRS Complex Detection			
Sensitivity	0.989	(0.978–0.999)	0.005
Precision	0.999	(0.998–0.999)	0.000
F1-Score	0.994	(0.988–0.999)	0.003
Weighted Averages			
PVC Precision	0.969	(0.924–0.993)	0.018
PVC Sensitivity	0.977	(0.960–0.990)	0.008
PAC Precision	0.928	(0.836–0.978)	0.040
PAC Sensitivity	0.714	(0.433–0.882)	0.122

Note: Weighted averages account for class imbalance in the dataset. Bootstrap confidence intervals computed using bias-corrected and accelerated (BCa) method.

## Data Availability

Public datasets employed in this study are accessible through the references provided in corresponding sections. Proprietary clinical data cannot be disseminated due to patient privacy regulations and contractual confidentiality agreements with data providers. The complete methodology, preprocessing pipeline, and algorithmic implementation are documented in sufficient detail to enable independent validation using comparable datasets.
